# Visualization of the Epiblast and Visceral Endodermal Cells Using *Fgf5-P2A-Venus* BAC Transgenic Mice and Epiblast Stem Cells

**DOI:** 10.1371/journal.pone.0159246

**Published:** 2016-07-13

**Authors:** Le Tran Phuc Khoa, Takuya Azami, Tomoyuki Tsukiyama, Jun Matsushita, Setsuko Tsukiyama-Fujii, Satoru Takahashi, Masatsugu Ema

**Affiliations:** 1 Department of Anatomy and Embryology, Ph.D. Program in Human Biology, School of Integrative and Global Majors, University of Tsukuba, Tsukuba, Ibaraki, Japan; 2 Department of Anatomy and Embryology, Faculty of Medicine, University of Tsukuba, Tsukuba, Ibaraki, Japan; 3 Department of Stem Cells and Human Disease Models, Research Center for Animal Life Science, Shiga University of Medical Science, Otsu, Shiga, Japan; 4 International Institute for Integrative Sleep Medicine, Life Science Center, University of Tsukuba, Tsukuba, Ibaraki, Japan; 5 Animal Resource Center, University of Tsukuba, Tsukuba, Ibaraki, Japan; 6 PRESTO, Japan Science and Technology Agency, Kawaguchi, Saitama, Japan; National University of Singapore, SINGAPORE

## Abstract

*Fibroblast growth factor 5* (*Fgf5*) has been widely used as a marker for the epiblast in the postimplantation embryo and epiblast stem cells (mEpiSCs) in the mouse, making it valuable for study of differentiation of various tissues and epiblast cells *in vivo* and *in vitro*. Here, we report for the first time the generation of *Fgf5-P2A-Venus* BAC transgenic (Tg) mice and show that the BAC Tg can recapitulate endogenous *Fgf5* expression in epiblast and visceral endodermal cells of E6.5 and 7.5 embryos. We also show that *Fgf5-P2A-Venus* BAC Tg mEpiSCs in the undifferentiated state expressed abundant Venus, and upon reprogramming into naïve state, Venus was suppressed. Furthermore, while most Tg mEpiSCs expressed Venus abundantly, surprisingly the Tg mEpiSCs contained a minor subpopulation of Venus-negative cells that were capable of conversion to Venus-positive cells, indicating that even *Fgf5* expression shows dynamic heterogeneity in mEpiSCs. Taken together, *Fgf5-P2A-Venus* BAC Tg mice and mEpiSCs generated in this study will be useful for developmental biology as well as stem cell biology research.

## Introduction

Mouse embryonic stem cells (mESCs) are the first pluripotent stem cell type that was derived from the inner cell mass of the developing blastocyst [1,2]. Self-renewal and pluripotency are the defining features of mESCs, meaning that these cells can be maintained indefinitely in culture while retaining their ability to differentiate into all cell lineages of an adult organism. It is well-known that the core pluripotency transcription factor network formed by *Oct3/4*, *Sox2*, and *Nanog* is connected with extracellular signaling pathways, such as leukemia inhibitory factor (LIF), bone morphogenetic protein, and Wnt, which shields mESCs from differentiating stimuli [3–5]. mESCs can be grown either in conventional medium supplemented with LIF and serum or in serum-free medium containing dual inhibitors (known as 2i) for mitogen activated protein kinase (Mapk) and glycogen synthase kinase-3 (Gsk3) [6].

Subsequent studies led to the establishment of another pluripotent stem cell type, termed epiblast stem cells (mEpiSCs), which are isolated from the postimplantation mouse epiblast [7,8]. Unlike mESCs whose pluripotency relies on LIF/Janus-associated kinase-signal transducer and activator of transcription 3 (Jak-Stat3) signaling, mEpiSC self-renewal is dependent on basic fibroblast growth factor (bFGF) and Activin/transforming growth factor beta (TGFβ) signaling. In addition, when injected back into the host blastocyst, mESCs highly contribute to chimera formation while only a very small fraction of mEpiSCs analogous to the early postimplantation epiblast can do so [9]. However, a recent study reported that mEpiSCs could readily form chimeras including germ cell lineage provided they were grafted to gastrulating embryos that retained pluripotency of the postimplantation epiblast [10]. Thus, the inherent discrepancies in colony morphology, molecular and epigenetic status and chimera formation support the notion that mESCs and mEpiSCs are representatives of distinct pluripotent states termed naïve and primed pluripotency, respectively [11]. Interestingly, these naïve and primed pluripotent states can be interconverted in defined culture conditions. Naïve mESCs can achieve a primed-like state by stimulating bFGF and Activin/TGFβ signaling while mEpiSCs can be reprogrammed back into a naïve-like state by a combination of 2i/LIF and forced expression of pluripotency-related factors, such as *Nanog*, *Esrrb*, *Klf2*, *Klf4* or *Klf5* [12–16].

Heterogeneity is an inherent feature of mESCs when grown in the conventional culture condition containing LIF and serum [17–20]. mEpiSCs also exhibit heterogeneous expression of *Oct3/4* and *T* (also known as *Brachyury*), resulting in differences in differentiation potential of subpopulations [9,21]. *Oct3/4*-negative mEpiSCs could not incorporate into the host blastocyst for chimera contribution, whereas a very small fraction of *Oct3/4*-positive mEpiSCs harboring distal enhancer activity of *Oct3/4* could efficiently form chimeras [9]. Furthermore, while *T*-positive mEpiSCs were prone to differentiate towards mesoderm and endoderm fates, a feature similar to that of *in vivo* epiblast cells that ingress through the primitive streak during gastrulation process, *T*-negative mEpiSCs had a propensity to give rise to the neuroectoderm cell lineage [21].

Fibroblast growth factors (FGFs) are structurally related proteins comprising 22 members in mammals [22]. The interactions between FGFs and FGF receptors (FGFRs) play important roles in regulating a wide variety of biological processes, ranging from modulation of tissue repair, inflammation, cell proliferation, survival and differentiation [23] to pluripotency and lineage specification [24] and regulation of energy expenditure [25]. Among the FGF family members, *Fgf5* is transiently expressed at different stages of the developing embryo [26]. Subsequent studies proposed a potential role of *Fgf5* in the process of gastrulation through stably maintaining the mobility of cells subjected to become the prospective embryonic germ layers [27–30]. *Fgf5* has since been used as a marker for epiblasts in pre-streak and streak stages of mouse embryos [31–33]. *Fgf5* is also strongly expressed in mEpiSCs [7,15,34,35], whereas it is hardly detectable in mESCs [36]. These findings implicated *Fgf5* as a valuable marker for differentiation study of various tissues and epiblast cells *in vivo* and *in vitro*. Therefore, generation of an animal model mimicking *Fgf5* expression *in vivo* and *in vitro* would be useful for a better understanding of epiblast cells and other biological events occurring during development as well as cell fate decision made by mEpiSCs.

Here we report for the first time the generation of *Fgf5-P2A-Venus* BAC (bacterial artificial chromosome) transgenic (Tg) mice to trace *Fgf5* expression during early embryonic development. Our results show the recapitulation of endogenous *Fgf5* expression governed by *Fgf5-P2A-Venus* BAC Tg in the postimplatation epiblast and visceral endodermal layer of E6.5 and E7.5 embryos as well as in mEpiSCs. Furthermore, surprisingly, while most Tg mEpiSCs expressed Venus abundantly, Tg mEpiSCs contained a minor subpopulation of Venus-negative cells that were capable of conversion to Venus-positive cells. This observation indicates that even *Fgf5* expression shows dynamic heterogeneity in mEpiSCs. These will serve as valuable tools for marking *Fgf5*-expressing cells during development and studying lineage commitment initiated by mEpiSCs.

## Material and Methods

### Construction of the *Fgf5-P2A-Venus* BAC Tg

The BAC clone (RP23-153I24) harboring the *Fgf5* gene was purchased from Invitrogen (Carlsbad, CA, USA). For generation of the reporter cassette, the *PGK-gb2-neo* sequence flanked by FRT sites was ligated to a DNA fragment encoding *P2A-Venus-ipacpA* (Gene Bridges, Heidelberg, Germany). The cassette was then inserted into the first or third exon of *Fgf5* by PCR amplification. The resulting BAC targeting vector and RED/ET expression plasmid (Gene Bridges) were co-transformed into *Escherichia coli*. After screening with Kanamycin, recombinants were identified by PCR analysis.

### Generation of Tg mice

Recombinant BAC DNAs were purified with a NucleobondXtra BAC Kits (Macherey-Nagel, Düren, Germany) and then linearized by Pl-SceI digestion. Pronuclear injection was performed in fertilized eggs isolated from C57B6/J females, followed by transplantation into pseudo-pregnant ICR females (SLC Inc., Shizuoka, Japan). Tg mice were confirmed by PCR genotyping with the following primer sequences: 5′-TTCAAGGACGACGGCAACTACAAGAC-3′ and 5′-GCTTCTCGTTGGGGTCTTTCTCAG-3′. The Tg mice were maintained on an ICR or B6 genetic background. This study was approved and conducted in accordance with the Regulations for Animal Experimentation of Shiga University of Medical Sciences.

### Immunohistochemical analysis

Mice were sacrificed by cervical dislocation. Embryos were then dissected, staged in accordance with Downs and Davies [37], and fixed in 4% paraformaldehyde for 30 min at 4°C. After washing twice in PBS, embryos were permeabilized in 0.5% TritonX-100 (Sigma-Aldrich, St. Louis, MO, USA) in PBS (0.5% TPBS) for 30 min at 4°C. Permeabilized embryos were then blocked in blocking solution containing 10% donkey serum (Immuno Bioscience, Mukilteo, WA, USA), 0.1% bovine serum albumin (Sigma-Aldrich) and 0.01% PBST (0.01% Tween20 in PBS, Nacalai Tesque, Inc., Kyoto, Japan) for 1 h at 4°C, followed by incubation overnight at 4°C with anti-Oct3/4 rabbit polyclonal antibody (1:300; Cat #Ab19857, Abcam, Cambridge, UK) and anti-Gata4 goat polyclonal antibody (1:300; Cat #sc-1237, Santa Cruz Biotechnology, Dallas, TX, USA) or anti-T goat polyclonal antibody (1:200; Cat #AF2085, R&D Systems, Minneapolis, MN, USA). After three washes with 0.5% TPBS, embryos were incubated with donkey anti-goat IgG Alexa-Fluor633-conjugated secondary antibody (1:500; Cat #A21082, Molecular Probes Inc., Eugene, OR, USA) and donkey anti-rabbit IgG Cy3-conjugated antibody (1:500; Cat #711-165-152, Jackson ImmunoResearch, West Grove, PA, USA) for 3 h at 4°C. Embryos were then washed three times with 0.5% TPBS and incubated with anti-GFP rabbit polyclonal antibody Alexa-Fluor488 conjugate (1:300; Cat #A21311, Molecular Probes Inc.) for 3 h at 4°C. Nuclei were stained with Hoechst33342 (2 μg/ml; Cat #H3570, Molecular Probes Inc.) for 20 min at 4°C. Images were captured using a Leica TCS-SP8 confocal microscope (Leica Microsystems, Wetzlar, Germany).

Immunohistochemical analysis for mESCs and mEpiSCs was performed as previously described [16].

### Whole-mount *in situ* hybridization

Fluorescent mRNA labeling by cytoplasmic fluorescence *in situ* hybridization (FISH) was performed as described previously [38]. The full-length coding region of mouse *Fgf5* was amplified by PCR from EpiSC cDNA using following primers: mFgf5 fwd, 5’-ATGAGCCTGTCCTTGCTCTTCCTC-3’ and mFgf5 rev, 5’-TCATCCAAAGCGAAACTTCAGTCTG-3’. Digoxigenin (DIG)-11-UTP (Cat# 11209256910, Roche) -labeled antisense RNA probes were generated by T7 RNA polymerase using SP6/T7 transcription kit (Cat# 10999644001, Roche). Hybridization with DIG-labeled probes was performed overnight at 65°C. The embryos were then incubated with a peroxidase conjugated anti-DIG antibody (Cat# 11207733910, Roche, Basel, Switzerland) for 1 h at room temperature. Fluorescent staining was carried out with a Tyramide signal amplification cyanine 3 system (TSA-Cy3) kit (Code# NEL704A, Perkin-Elmer, Waltham, MA, USA) according to the manufacturer’s recommendations. To amplify the fluorescence signal, a TSA-biotin amplification kit (Code# NEL700A, Perkin-Elmer) was used.

### Establishment of *Fgf5-P2A-Venus* BAC Tg mEpiSCs

*Fgf5-P2A-Venus* BAC Tg mEpiSCs were derived from E6.5 embryos (*Fgf5-P2A-Venus* BAC Tg male line #571 x ICR female) as described [8] with a minor modification in culture medium. We used NDiff227 (StemCells Inc., Newark, CA, USA) medium containing human Activin A (20 ng/ml; R&D Systems) and bFGF (12 ng/ml; Wako Pure Chemical Industries, Osaka, Japan).

### Cell culture

mESCs were maintained in ESC medium (DMEM supplemented with 10% fetal bovine serum (FBS), 1 mM sodium pyruvate, 0.1 mM 2-mercaptoethanol, 1X nonessential amino acids, 1 mM L-glutamine, 100 u/ml penicillin/streptomycin and 1000 U LIF per ml (prepared in house) on 0.1% gelatin-coated dishes and passaged every two days using 0.25% trypsin-EDTA as previously described [39].

### Cellular Reprogramming

To overexpress *Nanog* or *Klf5* in *Fgf5-P2A-Venus* BAC Tg mEpiSCs, we used piggyBac transposon and a transposase system. The pPB-CAG-Flox-*Nanog*/*Klf5*-dsRedT4-iresHygroR plasmid was generated by combination of the PB-CAG backbone and pPyCAG-Flox-*Nanog*/*Klf5*-dsRedT4-iresHygroR, both of which were kindly provided by Dr. Hitoshi Niwa (Kumamoto University, Japan). Plasmids were then co-transfected with piggyBac transposase into the Tg mEpiSCs as previously described [16]. After 7 days of selection with 250 μg/ml Hygromycin B (InvivoGen, San Diego, CA, USA), colonies were picked for stable *Klf5* and *Nanog*-overexpressing Tg mEpiSC lines.

For reprogramming experiments, 2–4 × 10^4^ cells were seeded onto fibronectin-coated 6-well plates in EpiSC culture conditions. After 24 h, the medium was switched to 2i/LIF conditions; the 2i inhibitors included 1 μM Mek inhibitor PD0325901 (Wako Pure Chemical Industries) and 3 μM Gsk3 inhibitor CHIR99021 (Wako Pure Chemical Industries). After 7 days, immunofluorescence analysis was performed. To check the characteristics of the resulting miPSCs, several miPSC colonies were picked up, expanded, and then cultured in 2i/LIF conditions for further experiments.

### Reverse transcription (RT)-quantitative (q) PCR analysis

Total RNAs were extracted using the RNeasy Micro Kit (Qiagen, Hilden, Germany), followed by cDNA synthesis using the ReverTra Ace (TOYOBO CO., LTD, Osaka, Japan) according to the manufacturer’s instructions. Real-time PCR was performed with the Thermal Cycler Dice Real Time System (Takara Bio Inc., Otsu, Shiga, Japan) and SYBR Premix EX Taq II (Takara Bio Inc.). Data were normalized against the expression of *β-actin* gene. Primer sequences are listed in S1 Table.

### Cell sorting

Cells were dissociated by 0.25% trypsin-EDTA and resuspended in DMEM supplemented with 10% FBS. To exclude dead cells, the single cell suspension was incubated with propidium iodide (Cat #P3566, Molecular Probes Inc.) for 10 min on ice. Flow cytometry analysis was performed with FACSCalibur (Becton Dickinson Biosciences, San Jose, CA, USA). Cell sorting was performed with a FACSAria Fusion (Becton Dickinson Biosciences).

### Statistical analysis

Student’s t-test was applied for statistical analysis. Data are presented as means and standard errors. Statistical significance was determined at *P* < 0.05.

## Results

### Generation of *Fgf5-P2A-Venus* BAC Tg mice

**Fig 1:**
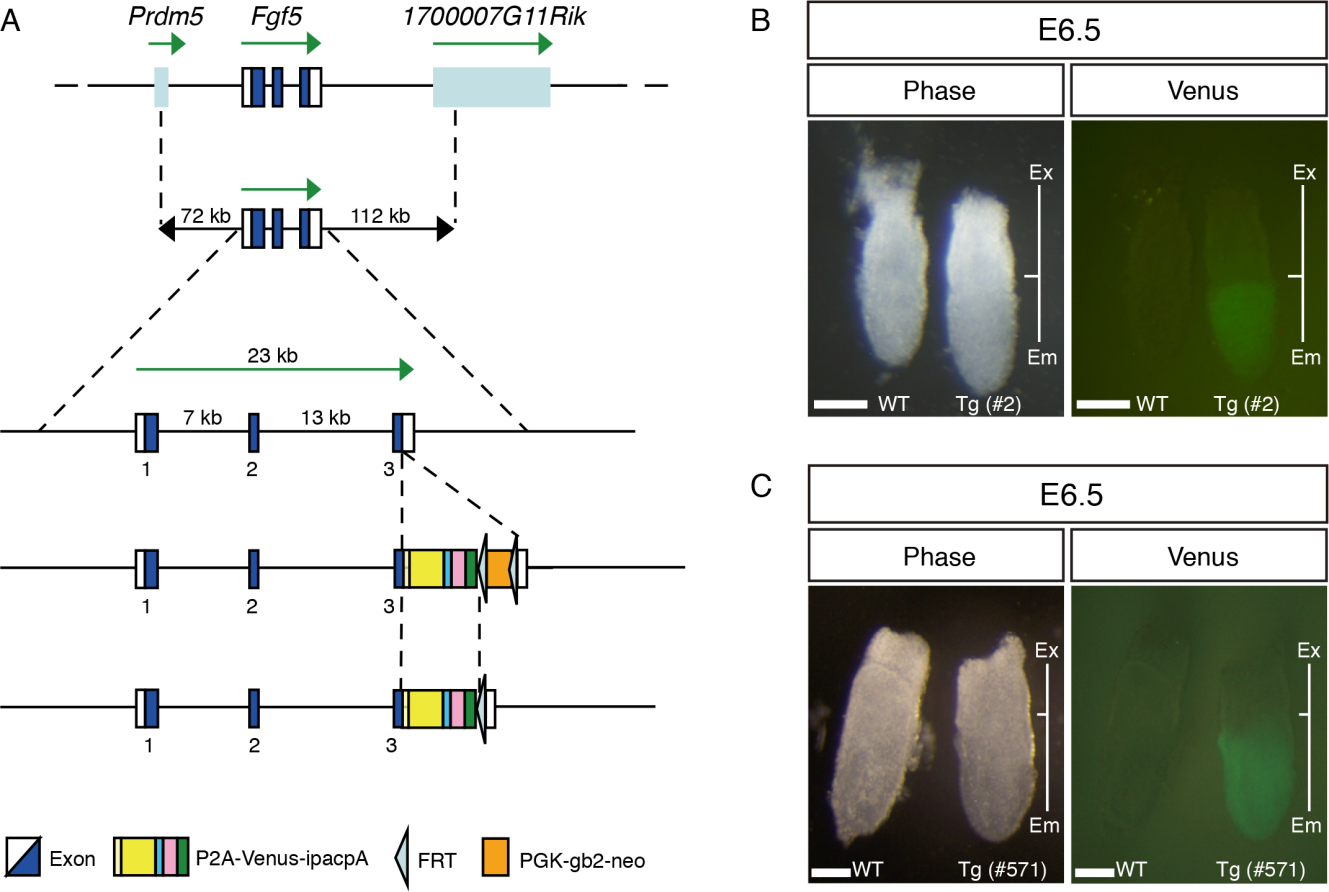
Generation of *Fgf5-P2A-Venus* BAC Tg mice. (A) Construction of the *Fgf5-P2A-Venus* BAC Tg. The *Fgf5* BAC clone (RP23-153I24) covering 72 kb upstream and 112 kb downstream of *Fgf5* gene was used. Note that the *PGK-gb2-neo* cassette was removed from the BAC construct prior to generation of Tg mice. P2A: porcine teschovirus-1 self-cleaving peptide; ipac: ires (internal ribosome entry site)-puromycin resistant gene. (B, C) Venus expression in WT and Tg embryos at E6.5 (line #2 and #571). Ex: extraembryonic region; Em: embryonic region. Scale bar: 100 μm.

To generate transgenic (Tg) mice recapitulating endogenous *Fgf5* expression, we took advantage of the enhanced yellow fluorescence protein Venus, which possesses valuable features for visualization, such as quick maturation and resistance to acidosis [40]. As the first trial, a BAC clone was used to cover the entire genomic region of *Fgf5* with a modification at the first exon in which an in-frame fusion Venus was implemented right after the start codon (S1 Fig). Although we obtained six Tg lines, Venus expression was not found in the epiblast of E6.5 and 7.5 embryos (data not shown). Because we anticipated that the reporter cassette perturbed potential regulatory regions located around the first exon and intron, we inserted the P2A (porcine teschovirus-1 self-cleaving peptide)-Venus reporter cassette into the third exon of *Fgf5* in the BAC clone (Fig 1A), and generated six lines of *Fgf5-P2A-Venus* BAC Tg mice. To check Venus expression, we dissected embryos at E6.5 and 7.5 from 6 Tg lines, and found strong Venus expression in the epiblast of all Tg lines (Fig 1B and 1C, data not shown), indicating that *Fgf5-P2A-Venus* BAC Tg construct can efficiently direct expression in the epiblast.

### Venus expression in *Fgf5-P2A-Venus* BAC Tg embryos

**Fig 2:**
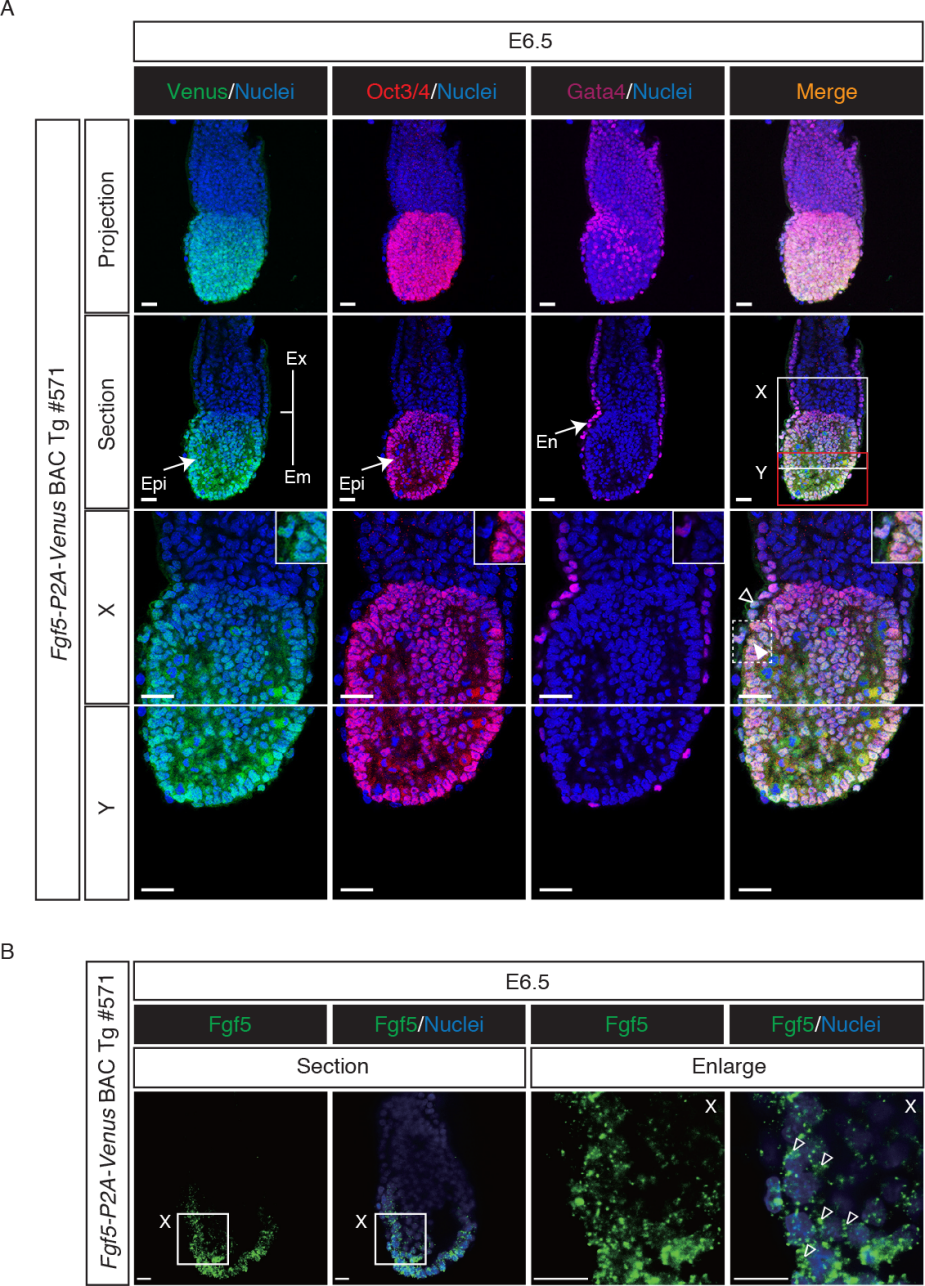
Venus expression in *Fgf5-P2A-Venus* BAC Tg embryo at E6.5. (A) Immunofluorescence analysis of the Tg embryo at E6.5 for Oct3/4 (red), Venus (anti-GFP, green), Gata4 (purple) and Nuclei (Hoechst33342, blue). Higher magnification of optical sections is shown in panels X and Y. Note that Oct3/4 and Venus were co-expressed in the epiblast of the Tg embryo, while Gata4 expression was specifically observed in the endoderm regions of the Tg embryo. Venus expression was also seen in visceral endodermal layer. Open and closed arrowheads indicate endodermal and epiblast cells, respectively. Ex: extraembryonic region; Em: embryonic region; Epi: epiblast; En: endoderm. Scale bar: 50 μm. All images were captured by a Leica TCS-SP8 confocal microscope using a 40x/1.25 oil objective lens. (B) Whole-mount fluorescence *in situ* hybridization of *Fgf5* in the Tg embryo at E6.5. Open arrowheads indicate cytoplasmic localization of endogenous *Fgf5* mRNA. Scale bar: 20 μm. Images were captured by a Leica TCS-SP8 confocal microscope using a 40x/1.25 oil objective lens.

**Fig 3:**
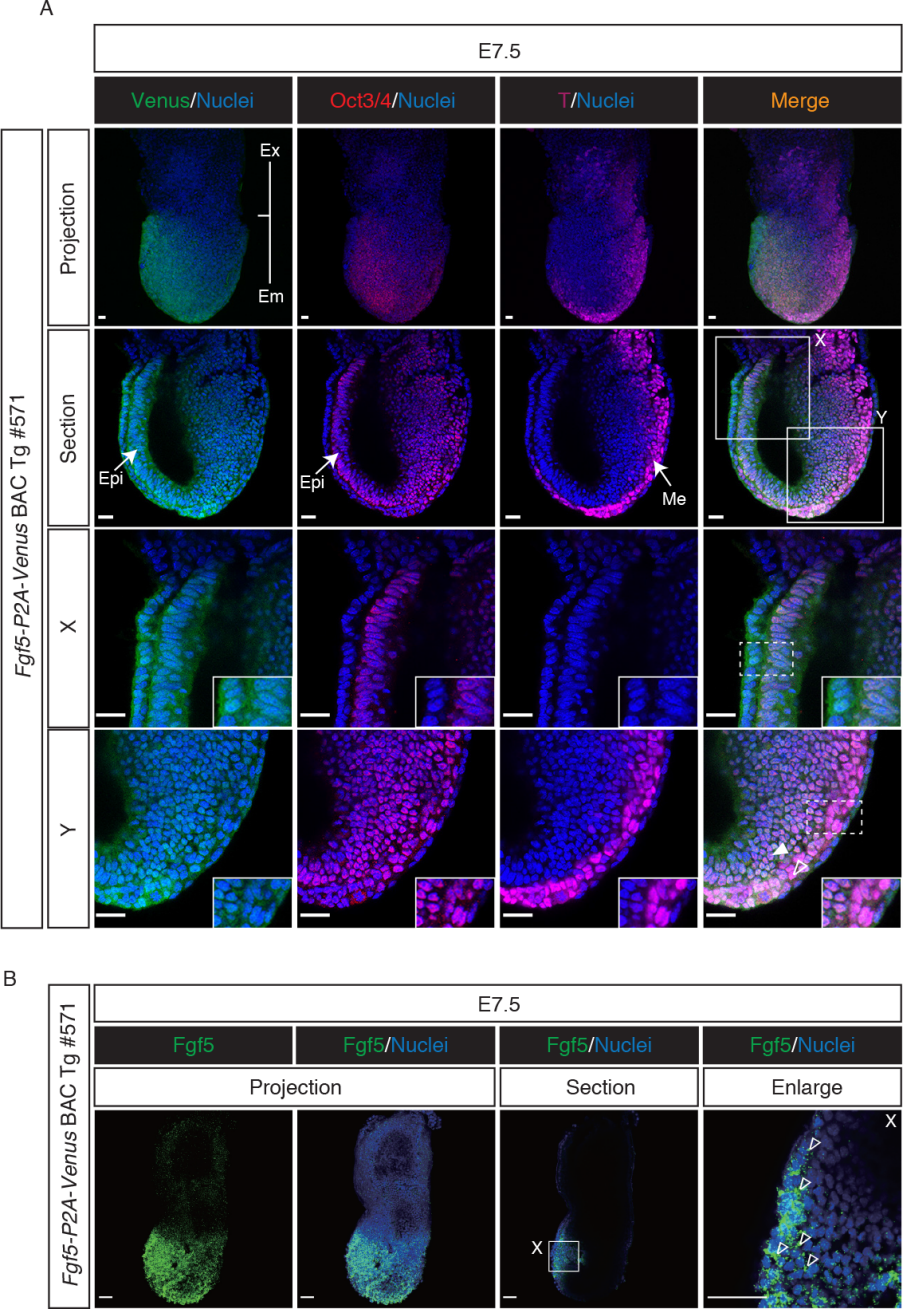
Venus expression in *Fgf5-P2A-Venus* BAC Tg embryo at E7.5. (A) Immunofluorescence images of the Tg embryo at E7.5 for Oct3/4 (red), Venus (anti-GFP, green), T (purple) and Nuclei (Hoechst33342, blue). Higher magnification of optical sections is shown in panels X and Y. Note that Oct3/4 and Venus were co-expressed in the epiblast of the Tg embryo, while T expression was observed in the mesodermal layer of the Tg embryo. Venus expression was also seen in visceral endodermal layer. Open and closed arrowheads indicate mesodermal and epiblast cells, respectively. Ex: extraembryonic region; Em: embryonic region; Epi: epiblast; Me: mesoderm; T: Brachyury/T. Scale bar: 50 μm. All images were captured by a Leica TCS-SP8 confocal microscope using a 20x/0.7 dry objective lens (projection images) and 40x/1.25 oil objective lens (section, X and Y images). (B) Whole-mount fluorescence *in situ* hybridization of *Fgf5* in the Tg embryo at E7.5. Open arrowheads indicate cytoplasmic localization of endogenous *Fgf5* mRNA. Scale bar: 50 μm.

The observation that Venus expression was detectable in the postimplantation epiblast of Tg embryos prompted us to examine detailed expression of Venus directed by the Tg. Embryos collected at E6.5 and E7.5 were stained for the epiblast marker Oct3/4 and endodermal marker Gata4 or mesodermal marker T (Figs 2 and 3). We found uniform expression of Venus in the epiblast of the Tg embryo at E6.5 (Fig 2A), consistent with previous reports [27–30]. Venus was also weakly seen in the visceral endodermal layer, in accordance with previous observations [27,28,30]. Higher magnification confirmed our observation that Venus and Oct3/4 expression were expressed uniformly throughout the epiblast, whereas Gata4 protein was only discernible in the visceral endoderm (Fig 2A). Importantly, fluorescent mRNA labeling by cytoplasmic FISH revealed the presence of endogenous *Fgf5* mRNA signals in the epiblast and visceral endodermal layer of Tg embryos at E6.5 (Fig 2B). These results indicated that the *Fgf5-P2A-Venus* BAC Tg construct recapitulated endogenous *Fgf5* expression in Tg embryos at E6.5. In Tg embryo at E7.5, Venus and Oct3/4 expression was found to be overlapping in the epiblast regions while Venus was also detected abundantly in the anterior visceral endoderm layer (Fig 3A), consistent with previous reports [28,41]. We also confirmed endogenous *Fgf5* mRNA expression in the epiblast and visceral endodermal layer of Tg embryos at E7.5 (Fig 3B). Collectively, these results demonstrated that *Fgf5-P2A-Venus* BAC Tg is capable of recapitulating endogenous *Fgf5* expression in the postimplantation epiblast and visceral endodermal layer.

While epiblast cells that ingress through the primitive streak can form the mesoderm and endoderm, epiblast cells that do not traverse the primitive streak can give rise to the ectoderm [42,43]. It is of note that at E8.25, we observed Venus expression in the neuroepithelium of the forebrain (S2 Fig), consistent with Venus expression in the anterior epiblast at E7.5.

### Derivation and characterization of *Fgf5-P2A-Venus* BAC Tg mEpiSCs

**Fig 4:**
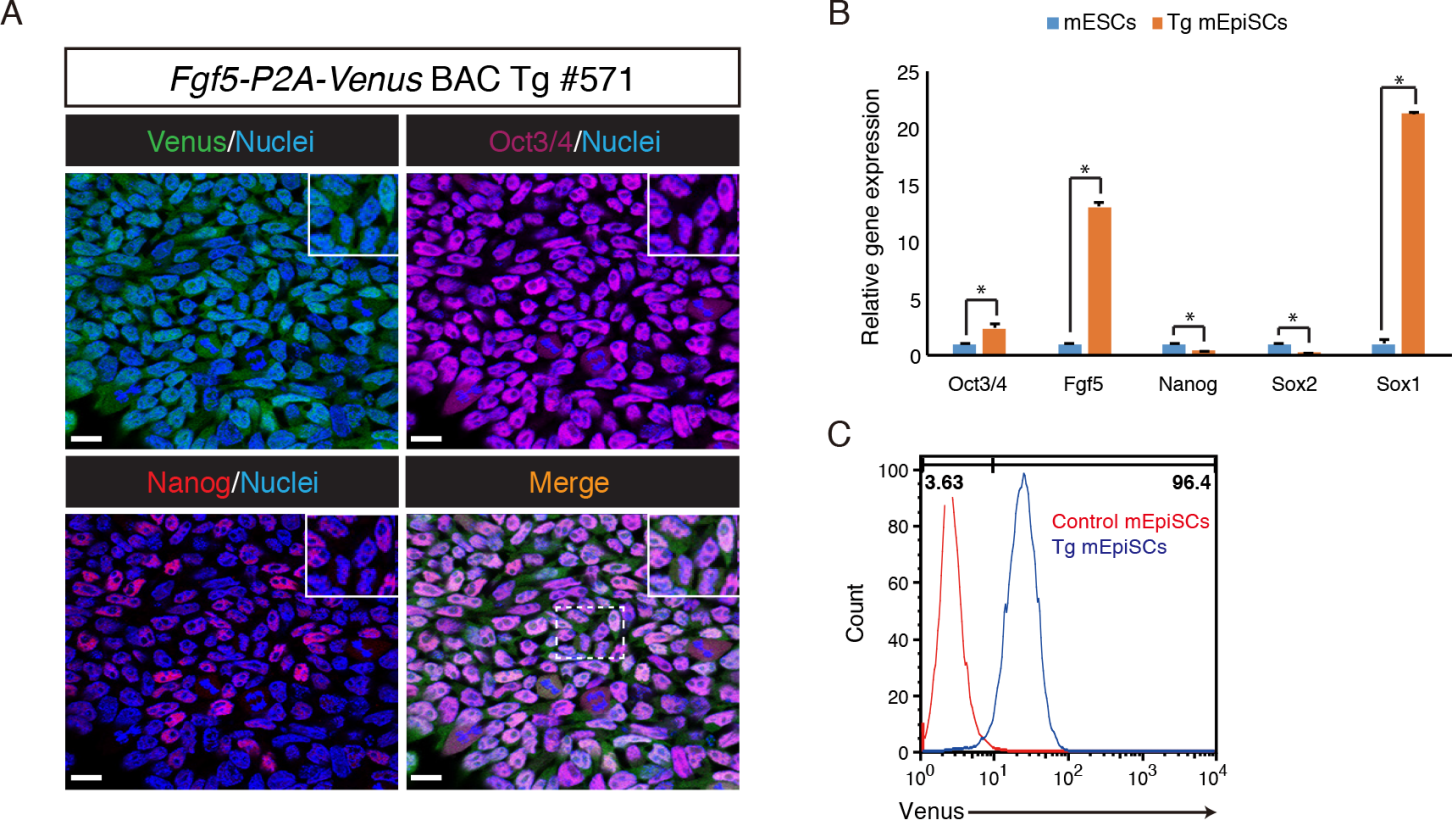
Derivation and characterization of *Fgf5-P2A-Venus* BAC Tg mEpiSCs. (A) Immunofluorescence analysis of the Tg mEpiSCs for Venus (anti-GFP, green), Oct3/4 (purple), Nanog (red) and Nuclei (Hoechst33342, blue). Scale bar: 20 μm. All images were captured by a Leica TCS-SP8 confocal microscope using a 63x/1.4 oil objective lens. (B) RT-qPCR analysis of genes associated with pluripotency and lineage commitment in the Tg mEpiSCs and mESCs. *β-actin* was used as endogenous control for normalization. The mean and SD of three independent experiments are shown. *P < 0.05. (C) Venus expression in control and the Tg mEpiSCs was analyzed by flow cytometry.

mEpiSCs represent a primed pluripotent state that can be utilized as a useful model for studying biological events that take place during the transition from the primed to naïve state and vice versa. To confirm the *Fgf5-P2A-Venus* BAC Tg expression *in vitro*, we established mEpiSCs, by culturing the epiblast of E6.5 *Fgf5-P2A-Venus* BAC Tg embryos in NDiff227 medium supplemented with bFGF and Activin. Immunofluorescence analysis revealed that the Tg mEpiSCs expressed pluripotency markers Oct3/4 and Nanog (Fig 4A). RT-qPCR analysis showed that *Oct3/4* expression is significantly higher in the Tg mEpiSCs than in mESCs, while expression levels of *Nanog* and *Sox2* were lower in the Tg mEpiSCs than in mESCs. Notably, *Fgf5* and *Sox1* expression were detected at much higher levels in the Tg mEpiSCs than in mESCs (Fig 4B). Flow cytometric analysis showed that most Tg mEpiSCs, if not all, expressed Venus, consistent with uniform Venus expression in the epiblast of the E6.5 Tg embryo (Fig 4C). Taken together, these results indicated that *Fgf5-P2A-Venus* BAC Tg mEpiSCs have similar properties to bona fide mEpiSCs.

### Reprogramming of *Fgf5-P2A-Venus* BAC Tg mEpiSCs into miPSCs

**Fig 5:**
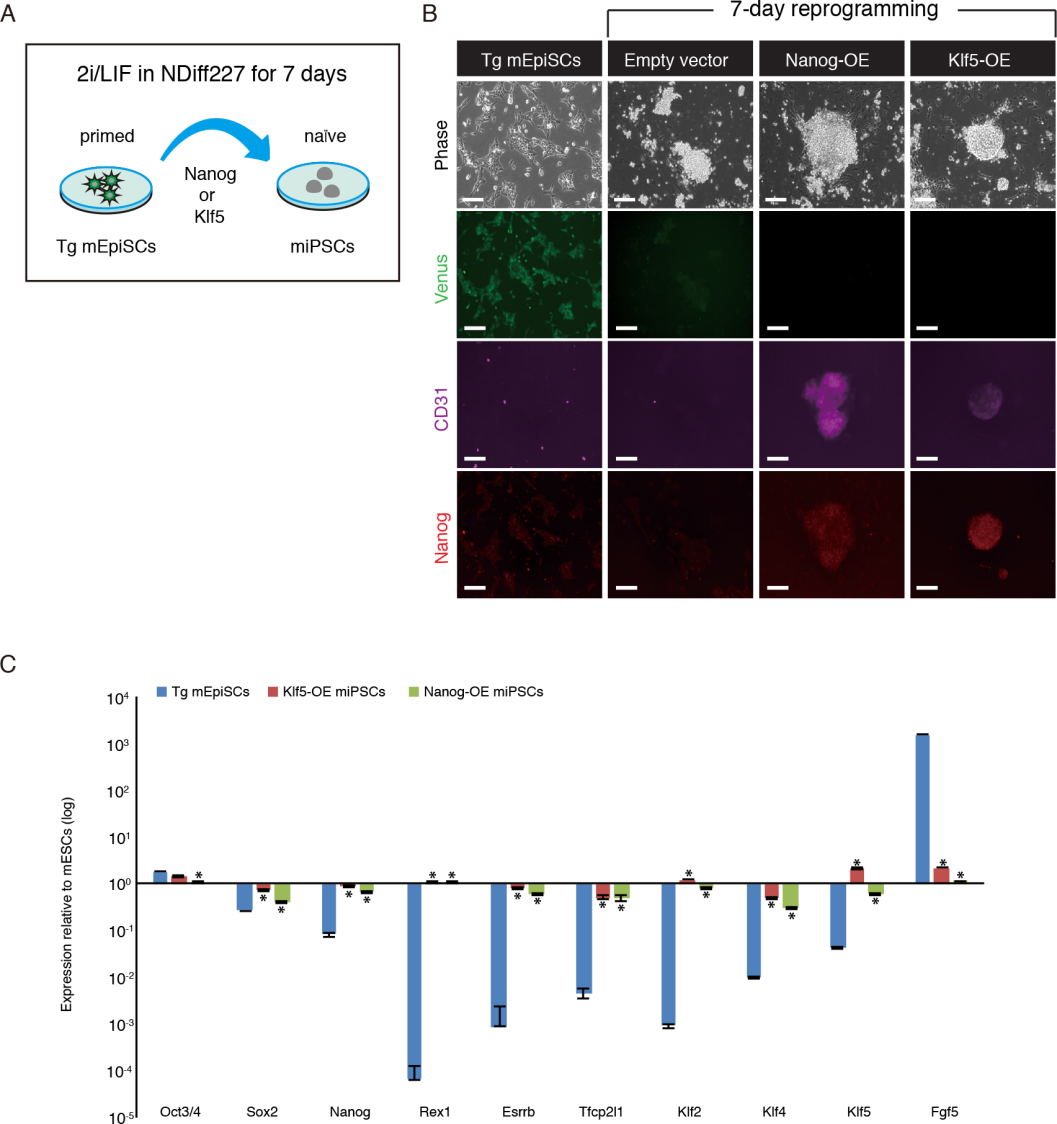
Reprogramming of *Fgf5-P2A-Venus* BAC Tg mEpiSCs into miPSCs. (A) Experimental scheme for reprogramming of the Tg mEpiSCs into miPSCs. The Tg mEpiSCs stably expressing *Klf5* or *Nanog* were cultured in NDiff227 medium supplemented with the Mek inhibitor (PD0325901), Gsk3 inhibitor (CHIR99021) and LIF. After 7 days, miPSC colonies were subjected to immunostaining. (B) Immunostaining for Venus (anti-GFP, green), CD31 (purple) and Nanog (red) in untransfected, vector control and miPSCs. OE: overexpression. Scale bar: 100 μm. (C) RT-qPCR analysis of Tg mEpiSCs and miPSCs. The mean and SD of two independent experiments are shown. *P < 0.05.

Reprogramming of mEpiSCs into miPSCs can be accomplished by ectopic expression of *Nanog*, *Esrrb*, *Klf2*, *Klf4* or *Klf5* cultured in the presence of 2i/LIF. [12–16]. The reprogramming process upregulates many naïve state-associated markers containing *Nanog*, *Esrrb*, *Tfcp2l1*, *Cd31*, *Rex1*, *Stella*, *Nr0b1*, *Prdm14*, *Nr5a2*, *Tbx3*, *Klf2*, *Klf4* and *Klf5*, with a parallel rapid reduction in the expression of primed state-associated markers such as *Fgf5*. Next, we asked whether Venus fluorescence is suppressed during the reprogramming process toward miPSC state. After ectopic expression of *Nanog* or *Klf5* in the Tg mEpiSCs, the culture medium was changed from bFGF and Activin to 2i/LIF (Fig 5A). We found that ES-like colonies emerged in the *Klf5*- and *Nanog*-overexpressing Tg mEpiSCs within 5–7 days after addition of reprogramming medium. Immunofluorescence analysis revealed that overexpression of *Klf5* or *Nanog* could reactivate the expression of CD31 (also known as PECAM-1: platelet endothelial cell adhesion molecule-1), a useful marker of inner cell mass cells, and increase Nanog expression in the miPSCs (Fig 5B). Importantly, Venus expression in the Tg mEpiSCs was markedly reduced in the miPSCs (Fig 5B). To further validate the characteristics of the miPSCs, several miPSC colonies were randomly picked to generate miPSC lines. These miPSC lines were maintained in 2i/LIF conditions and used for further experiments. RT-qPCR analysis showed upregulation of pluripotency factors *Nanog*, *Rex1*, *Esrrb*, *Tfcp2l1*, *Klf2*, *Klf4*, *and Klf5*, and downregulation of lineage commitment factor *Fgf5* in miPSCs (Fig 5C). Taken together, these results demonstrated that Venus expression can be used as an indicator when the Tg mEpiSCs are forced to reprogram into miPSCs.

### Dynamic heterogeneity of *Fgf5* expression in *Fgf5-P2A-Venus* BAC Tg mEpiSCs

**Fig 6:**
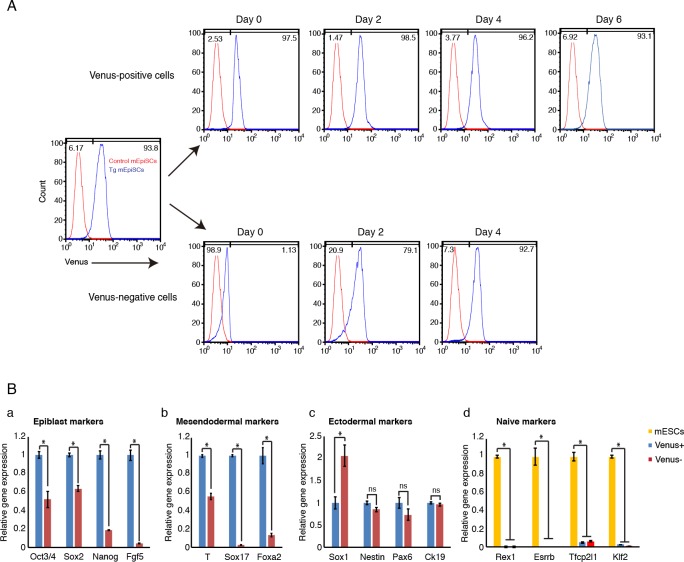
Dynamic heterogeneity of *Fgf5* expression in *Fgf5-P2A-Venus* BAC Tg mEpiSCs. (A) Venus-positive and -negative populations were purified, cultured and subjected to flow cytometry analysis at indicated days. Note that Venus-positive and -negative cells were interchangeable within 48 h post culture, and Venus-positive and -negative cells could re-establish the original cell state within 6 and 4 days, respectively. (B) Gene expression was examined by RT-qPCR in Venus-positive and -negative cells. The mean and SD of three independent experiments are shown. *P < 0.05.

mEpiSCs consist of several subpopulations: *T*-positive and -negative populations, and also *Sox1*-positive and -negative populations; these positive/negative populations are interconverted [21]. Although we found that *Fgf5-P2A-Venus* BAC Tg embryo showed uniform Venus expression in the epiblast and most Tg mEpiSCs expressed Venus abundantly, we investigated whether the Tg mEpiSCs contained a Venus-negative population. Careful examination of flow cytometry revealed that a minor fraction of the Tg mEpiSCs was Venus-negative (about 4%) (Fig 4C). To further explore this phenomenon, we purified Venus-positive and -negative mEpiSCs by cell sorting (Fig 6A) and cultured each cell fraction in mEpiSC growth conditions to investigate the ability to re-establish heterogeneity from each subpopulation. Interestingly, Venus-positive cells quickly emerged from the sorted Venus-negative cells, and the sorted Venus-negative cells could re-establish the original cell state within 4 days in culture (Fig 6A). Similarly, the purified Venus-positive cells also generated Venus-negative cells, although the re-establishment of heterogeneity from Venus-positive cells occurred more slowly compared with that from Venus-negative cells (Fig 6A). These results indicated that the Tg mEpiSCs contain at least two subpopulations that can be interconverted. We also examined gene expression patterns in Venus-positive and -negative mEpiSCs by RT-qPCR analysis (Fig 6B). We confirmed that *Fgf5* mRNA expression was enriched in Venus-positive cells (Fig 6Ba). Importantly, we found that the expression levels of other epiblast markers, *Oct3/4*, *Nanog*, and *Sox2*, were predominantly detectable in Venus-positive cells (Fig 6Ba). Furthermore, both subpopulations expressed very low levels of naïve pluripotency markers *Rex1*, *Esrrb*, *Tfcp2l1*, and *Klf2* compared with mESCs (Fig 6Bd). Interestingly, we found that mesendodermal markers *T*, *Sox17* and *Foxa2* were expressed at significantly higher levels in Venus-positive cells than in Venus-negative cells (Fig 6Bb). On the other hand, among the tested ectodermal markers, only *Sox1* expression was enriched in Venus-negative cells relative to Venus-positive cells (Fig 6Bc). Taken together, while it was thought that *Fgf5* marks mEpiSCs uniformly, our experiments clearly demonstrated that even *Fgf5* expression shows dynamic heterogeneity in mEpiSCs.

## Discussion

In this study, we demonstrated that *Fgf5-P2A-Venus* BAC Tg mice can recapitulate endogenous *Fgf5* expression in the postimplantation epiblast and visceral endoderm. To the best of our knowledge, this is the first Tg mouse model allowing for the visualization of endogenous *Fgf5* expression during early embryonic development. Initial Tg lines carrying Venus at the first exon failed to drive epiblast Venus expression while Tg lines carrying P2A-Venus in the place of the stop codon drove strong Venus expression in the epiblast of all Tg lines. The exact reason underlying these observations is currently not clear. Because many genes harbor important regulatory elements around the first intron, the insertion of the Venus-pA reporter cassette into the first exon could have potentially abolished transcription by perturbing the epiblast promoter/enhancer elements.

Previous studies showed that primitive streak formation in the posterior portion of epiblast is a crucial event through which the body plan is established during the gastrulation process [44]. More specifically, while epiblast cells that ingress through the primitive streak can form the mesoderm and endoderm, epiblast cells that do not traverse the primitive streak can give rise to the ectoderm [42,43]. Future work will delineate the actual cell fates of *Fgf5*-positive epiblast cells, which would provide important insights into how the ectoderm lineage is established and regulated in the gastrulating mouse embryo.

Gastrulation begins when a population of epiblast cells is triggered to move to the primitive streak located in the posterior epiblast while the other cells remain in the epiblast. This cell movement leads to the formation of the primary germ layers, namely the ectoderm, mesoderm, and endoderm [43,44]. However, how epiblast cell movement is regulated and which factors stimulate and determine the fates of cell populations at the onset of gastrulation is not fully understood. Therefore, a detailed analysis of *Fgf5* and *T* expression pattern will be required to understand the molecular basis of epiblast cell behavior during gastrulation.

mESCs consist of several subpopulations; the subpopulations expressing either PECAM1, Rex1 or Stella efficiently form chimeric animals when injected into blastocysts. mEpiSCs are also heterogeneous in terms of gene expression: *T*-positive cells are primed to differentiate into mesoderm and endoderm lineages, while *T*-negative cells are primed to ectoderm [21]. Our data showed that *Fgf5* overall uniformly marks mEpiSCs, consistent with the previous report [21]. However, surprisingly, there is a small subpopulation of *Fgf5*-negative cells in mEpiSCs. Our gene expression analysis revealed that, while *Fgf5*-positive cells predominantly expressed important mesendodermal markers such as *T*, *Sox17* and *Foxa2*, *Fgf5*-negative cells exhibited a high expression level of the ectodermal marker *Sox1*. Currently, the actual cell type of *Fgf5*-Venus-negative cells is unknown, but these results suggest the possibility that the heterogeneous expression of *Fgf5* observed in our study may be the foundation for the distinct differentiation biases of subpopulations in mEpiSCs. Thus, investigation of the differentiation propensity of *Fgf5*-negative and -positive mEpiSCs in response to differentiation stimuli will be of a great interest in future studies.

Previous studies indicated potential roles of *Fgf5* in the progression of hepatic fibrosis [45], the process of gastrulation [27,28] and hair growth cycle [46], but the molecular basis of how *Fgf5* manifests its functions has not been clearly understood. In addition, the impact of biological events on lineage commitment initiated by mEpiSCs is not known. Taken together, *Fgf5-P2A-Venus* BAC Tg mice and mEpiSCs established in our study may be used to investigate novel functions of *Fgf5* as well as to unravel molecular mechanisms underlying lineage specification *in vivo* and *in vitro*.

## Supporting Information

S1 Fig Construction of the first exon *Fgf5-Venus* BAC Tg. The *Fgf5* BAC clone (RP23-153I24) covering 72 kb upstream and 112 kb downstream of *Fgf5* gene was used. Note that Venus was fused in frame after the start codon.
(TIF)

S2 Fig Venus expression in *Fgf5-P2A-Venus* BAC Tg embryos at E8.25. Immunofluorescence staining of the Tg embryo at E8.25 for Venus (green). Note that Venus was expressed in the neuroepithelium of the Tg embryo. Ne: neuroepithelium. Scale bar: 100 μm.
(TIF)

S1 Table Primers used for RT-qPCR analysis. (TIF)
