# Supplementary figures and images for: Visualization of the Epiblast and Visceral Endodermal Cells Using Fgf5-P2A-Venus BAC Transgenic Mice and Epiblast Stem Cells
Source: PLoS One. 2016 Jul 13;11(7):e0159246. doi: 10.1371/journal.pone.0159246 (PMC4943650; doi:10.1371/journal.pone.0159246)

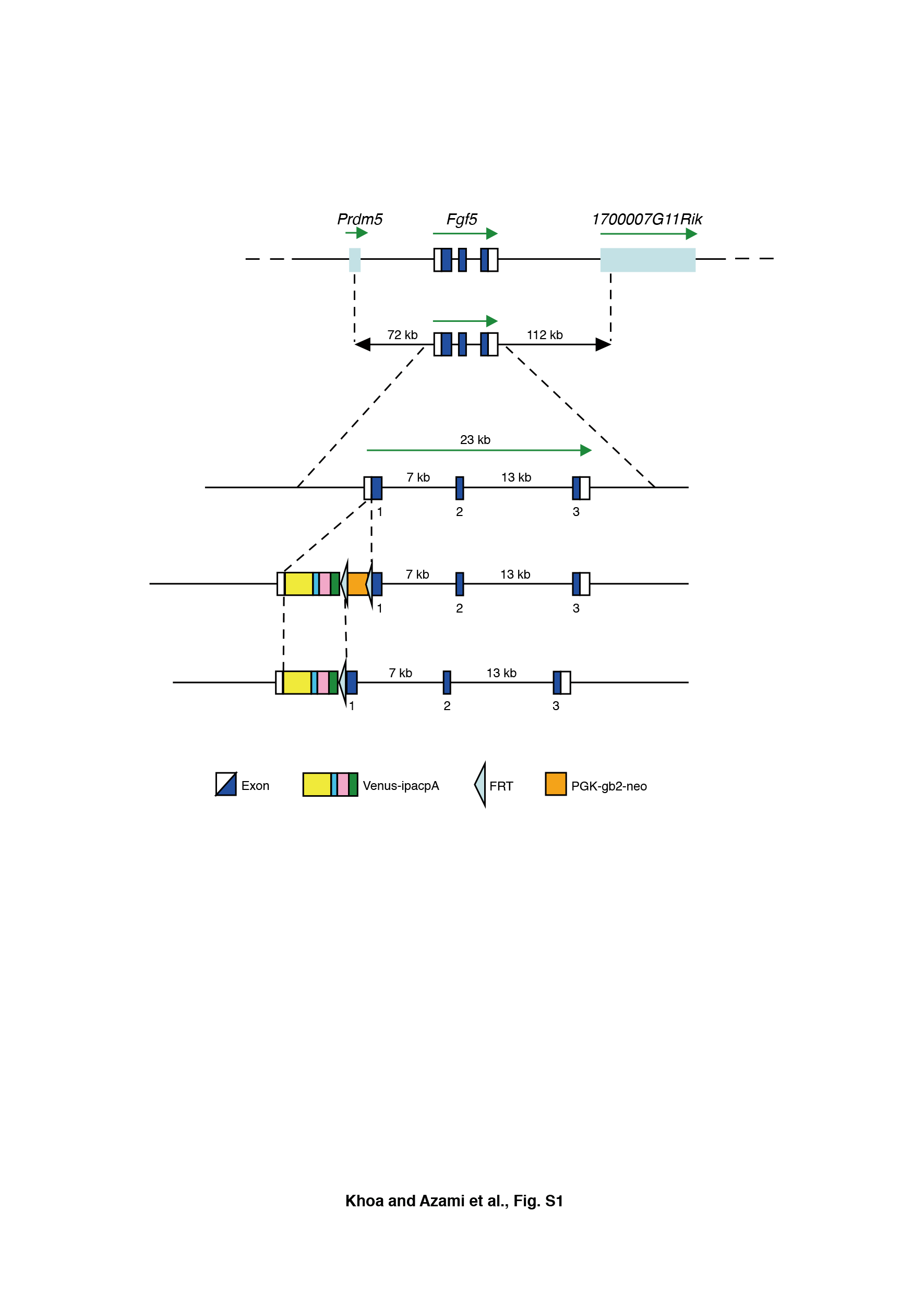

Supplement: S1 Fig — The Fgf5 BAC clone (RP23-153I24) covering 72 kb upstream and 112 kb downstream of Fgf5 gene was used. Note that Venus was fused in frame after the start codon. (TIF) [file pone.0159246.s001.tif]

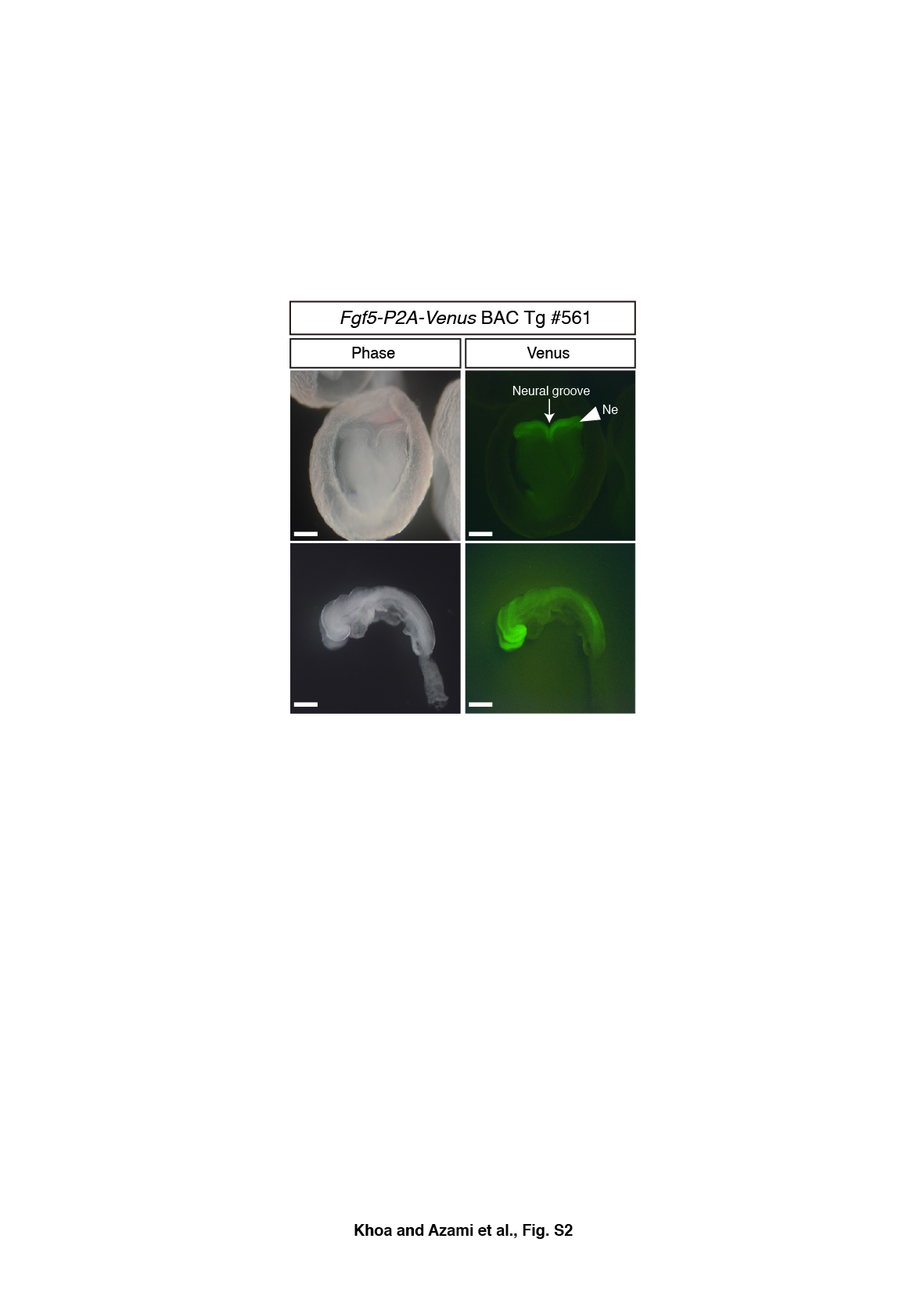

Supplement: S2 Fig — Immunofluorescence staining of the Tg embryo at E8.25 for Venus (green). Note that Venus was expressed in the neuroepithelium of the Tg embryo. Ne: neuroepithelium. Scale bar: 100 μm. (TIF) [file pone.0159246.s002.tif]

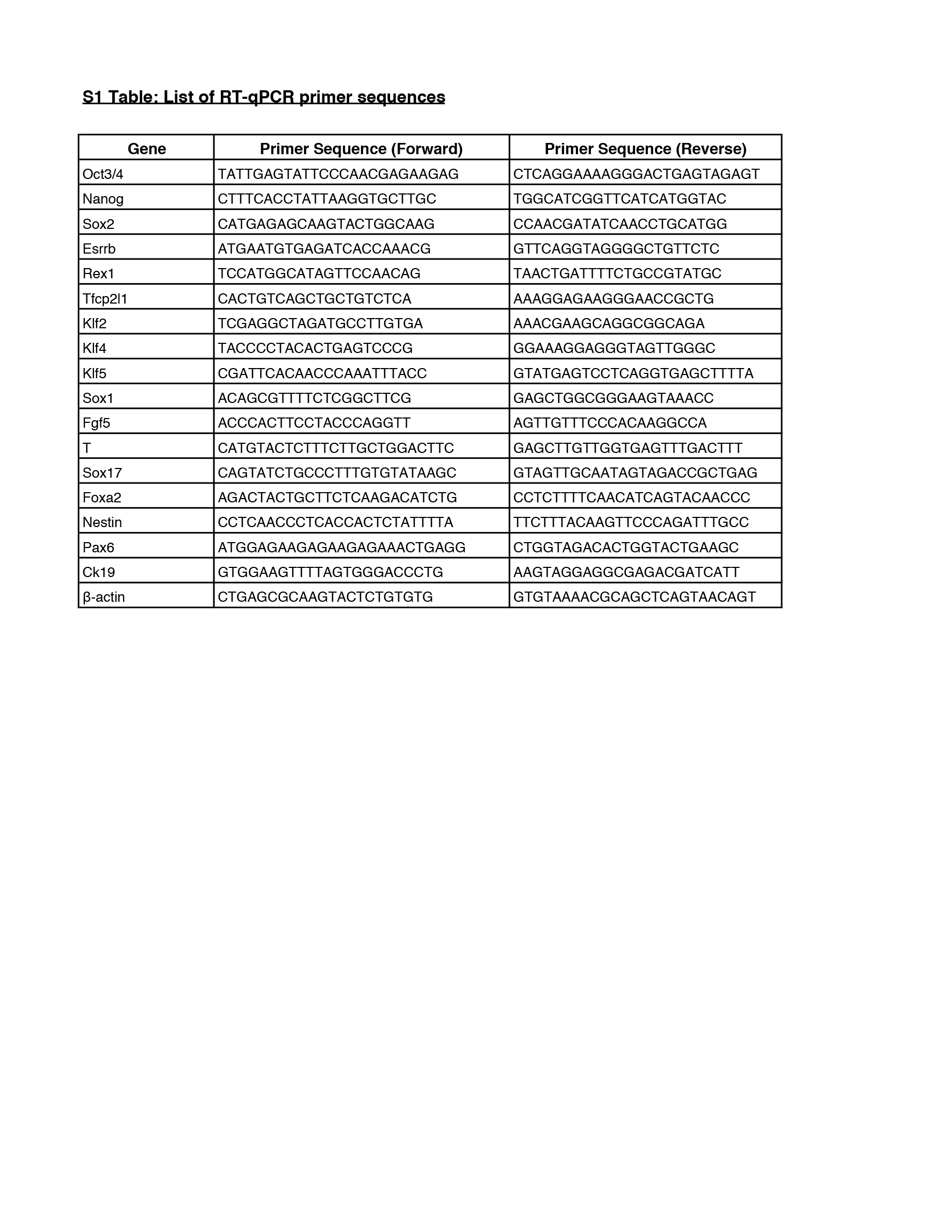

Supplement: S1 Table — (TIF) [file pone.0159246.s003.tif]
